# Diagnosing vascular cognitive impairment: Current challenges and future perspectives

**DOI:** 10.1177/17474930211073387

**Published:** 2022-01-30

**Authors:** J Matthijs Biesbroek, Geert Jan Biessels

**Affiliations:** 1 Department of Neurology, UMC Utrecht Brain Center, University Medical Center Utrecht, Utrecht, The Netherlands; 2 Department of Neurology, Diakonessenhuis Hospital, Utrecht, The Netherlands

**Keywords:** Vascular dementia, small vessels disease, diagnostic criteria, brain imaging

## Abstract

Cerebrovascular disease is a major cause of cognitive decline and dementia. This is referred to as vascular cognitive impairment (VCI). Diagnosing VCI is important, among others to optimize treatment to prevent further vascular injury. This narrative review addresses challenges in current diagnostic approaches to VCI and potential future developments. First we summarize how diagnostic criteria for VCI evolved over time. We then highlight challenges in diagnosing VCI in clinical practice: assessment of severity of vascular brain injury on brain imaging is often imprecise and the relation between vascular lesion burden and cognitive functioning shows high intersubject variability. This can make it difficult to establish causality in individual patients. Moreover, because VCI is essentially an umbrella term, it lacks specificity on disease mechanisms, prognosis, and treatment. We see the need for a fundamentally different approach to diagnosing VCI, which should be more dimensional, including multimodal quantitative assessment of injury, with more accurate estimation of cognitive impact, and include biological definitions of disease that can support further development of targeted treatment. Recent developments in the field that can form the basis of such an approach are discussed.

## Introduction

Cerebrovascular disease is the second most common cause of dementia, after Alzheimer’s disease.^1,2^ It is important to recognize and diagnose vascular contributions to cognitive impairment, among others to provide individualized treatment to prevent further vascular injury. This short review addresses diagnostic criteria for so-called “vascular cognitive impairment” (VCI). We will reflect on why it has proven difficult to capture VCI in a single diagnostic construct that informs on disease mechanisms, prognosis, and treatment; identify challenges in applying current VCI criteria in clinical practice; and discuss potential opportunities to update VCI criteria in light of recent developments in the field.

## Evolution of diagnostic constructs

Vascular factors in dementia already featured prominently in early descriptions by Otto Binswanger and Alois Alzheimer at the turn of the 20th century, at that time largely based on neuropathology. The first formal diagnostic criteria for vascular contributions to cognitive impairment appeared some 30 years ago^3,4^ and several iterations have appeared since.^1,2,5–7^ Here we discuss these diagnostic constructs under the umbrella term VCI; acquired cognitive impairment attributed to cerebrovascular disease. Essentially, all VCI criteria entail three basic components: there should be acquired cognitive impairment, there should be cerebrovascular disease, and the two should be causally related. Yet, there are fundamental differences in how these components have been operationalized. Initial criteria only considered dementia,^3,4^ an advanced stage of cognitive impairment where activities of daily life are affected. To accommodate the full spectrum of cognitive changes associated with vascular injury, also permitting diagnoses in earlier stages of disease, current VCI criteria encompass any degree of acquired cognitive impairment that can be objectified with cognitive testing.^5–7^ All criteria are generally inclusive regarding types of cerebrovascular disease considered, including ischemic (both of arterial or venous origin) and hemorrhagic injury, large and small vessel disease, and considering emboli, vasculopathies, and hypoperfusion.^1,3,4,7^ This inclusiveness clearly limits specificity in terms of disease mechanisms, but also in prognostic value and guidance for treatment. Where initial criteria aimed to capture “pure vascular dementia,” where no other pathologies explaining the cognitive deficit should be present,^3^ most criteria now acknowledge that cerebrovascular disease often co-occurs with other pathologies and that mixed pathologies need to be considered.^5–7^ Indeed, mixed pathologies are the rule rather than an exception in people with cognitive impairment, particularly at older age.^8^ Even among patients assumed to have pure vascular dementia, a substantial subset also has biomarker evidence of co-occurring Alzheimer pathology.^9^ The third component of the diagnosis, attributing cognitive impairment to cerebrovascular disease, shows considerable variation between criteria. When there is a clear temporal relationship between the occurrence of one or multiple cerebrovascular events and the onset of impairment, causality may be self-evident. This may be even more clear if symptomatic lesions involve locations known to predispose to cognitive impairment, also referred to as strategic lesions.^5,10^ However, the majority of people with cognitive impairment have so-called covert cerebrovascular disease, not manifested in a history of stroke. Because this covert disease is common also among older people without cognitive impairment, it has proven challenging to define the actual burden of vascular injury that can be accepted as cause of cognitive impairment. Finally, an emerging issue in VCI is that of cerebral reserve capacity: the functional impact of vascular injury is likely also determined by the resilience of the brain.^11^ The latter is a construct that has proven difficult to operationalize and is not yet considered in diagnostic criteria.

Maybe we should take a step back and reflect on what the actual purpose of diagnostic criteria for VCI should be? Separate vascular etiologies from other non-vascular etiologies? In light of the common occurrence of mixed pathologies, this may be futile. Claim the biggest possible chunk of the “dementia causality pie”? The question is if this is a real service to the field, as “vascular” is not an etiological entity and heterogeneous in terms of mechanism and treatment. And are criteria primarily meant for clinical practice or for research, in particular clinical trials? Of note, the actual treatment of the vascular disease is generally not determined by its cognitive impact. Take, for example, a patient with a lacunar ischemic stroke without cognitive deficits, another patient with a strategic lacunar stroke in the left thalamus causing cognitive impairment, and another patient with pre-existent dementia due to Alzheimer’s disease with a lacunar stroke. Stroke prevention strategies would likely be the same in all cases. A final point to consider is that diagnostic criteria for VCI tend to categorize both cognitive impairment and vascular injury in terms of presence or absence. These dichotomizations may help to create a language for communication with patients and among professionals, but also tend to create biological and conceptual silos that do not match with reality.

## Diagnosing VCI in clinical practice

The next sections briefly illustrate application of current diagnostic constructs for VCI in clinical practice, identifying challenges, but also providing some practical solutions (Summarized in Box 1).

**Box 1.** Stepwise approach to diagnosing and treating VCI.

1. Assess clinical symptoms ● Cognitive domains and neuropsychiatric symptoms ● Other neurological signs: focal deficits, gait disorder, urinary symptoms ● Impact on activities of daily living ● Vascular risk factors 2. Assess vascular brain injury ● Identify history of stroke and vascular brain lesions ● Determine if the lesion burden is more than expected relative to the patient’s age ● If possible, classify etiology of vascular lesions 3. Relate vascular brain lesions to clinical profile ● Match clinical symptoms with burden and location of lesions ● In case of history of stroke: establish temporal relation with symptom onset 4. Treatment ● General symptomatic treatment ● No prior vascular event: treat vascular risk factors (primary prevention guidelines) ● In case of stroke or other manifest cardiovascular event: secondary prevention guidelines generally apply

VCI: vascular cognitive impairment.

### Assessment of cognitive symptoms

A careful history taking from the patient and a knowledgeable informant is critical to determine the nature of cognitive symptoms, their impact on activities of daily living, and their course of development over time (i.e. gradual progression, stepwise decline, temporal relation with vascular events). Attention should also be paid to possible neuropsychiatric symptoms, gait disturbances, focal deficits on neurological examination, and urinary incontinence. Modifiable vascular risk factors (i.e. hypertension, diabetes mellitus, smoking, hyperlipidemia, obesity, excessive alcohol consumption, poor diet, inadequate exercise)^12^ should be systematically recorded.

Cognitive manifestations of VCI are heterogeneous. Traditionally, pronounced mental slowing and executive dysfunction combined with gait impairment and urinary incontinence is regarded as the typical phenotype of VCI. However, all cognitive domains can be affected, likely also depending on the nature and location of vascular brain injury.^13^ Cognitive evaluation using bedside tests and/or cognitive screening instruments should therefore address multiple cognitive domains. The Montreal Cognitive Assessment (MoCA) has been developed to this end.^14^ If one uses tests like the mini-mental state examination (MMSE), with low sensitivity to detect executive dysfunction,^14^ this domain should be probed with additional tests such as the frontal assessment battery (FAB).^15^ It should be kept in mind that processing speed is not sufficiently captured by any of these tests. A more detailed neuropsychological assessment may be indicated in case of discrepancies between cognitive complaints and the initial brief cognitive assessment or when the expected deficits are relatively subtle in nature.

### Assessment of vascular brain injury and disease mechanisms

**Figure 1.**
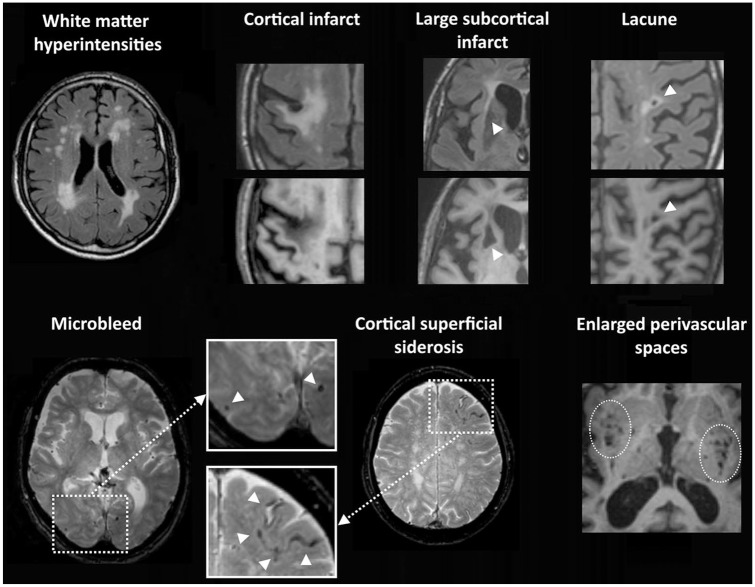
Vascular lesion types on brain MRI. Top row: white matter hyperintensities are visible as high signal on the Fluid Attenuated Inversion Recovery (FLAIR) image. Chronic cortical infarcts, large subcortical infarcts, and lacunes of presumed vascular origin are visible as fluid-filled cavities (hypo-intense on the T1 image) with surrounding gliosis (hyperintense signal on the FLAIR image). Bottom row: microbleeds are visible as small sphere-shaped hypo-intense lesions, and cortical superficial siderosis as a hypo-intense rim along the cortical surface on T2*-weighed images. Perivascular spaces in the basal ganglia and subinsular regions are shown as groups of small fluid-filled cavities that follow the orientation of penetrating vessels on this T1-image.

Brain imaging forms the cornerstone to establish the nature and severity of vascular brain injury. Vascular lesions types that can be detected on brain magnetic resonance imaging (MRI) are summarized in Figure 1. MRI is preferred over computed tomography (CT) due to the higher sensitivity for detecting small brain lesions, white matter hyperintensities (WMH), and the ability to detect microbleeds and superficial siderosis. In case of MRI contra-indications or unavailability, CT is adequate for ruling out alternative causes of cognitive impairment (e.g. subdural hematoma, brain tumor) and for demonstrating large infarcts, most lacunes, severe white matter lesions, and brain atrophy.^12,16^ The burden of brain atrophy and vascular brain lesions should be weighted according to the patient’s age. In the general population, the prevalence of WMH increases from 50% at 45 years to 95% at 80 years of age.^17,18^ Silent brain infarcts, lacunes, microbleeds, pronounced perivascular spaces, and brain atrophy are also commonly found in asymptomatic individuals.^12,18^ A known dilemma in assessing the burden of vascular brain injury, particularly for small vessel disease, is that normative data are not routinely available in a clinical setting and generally not part of radiology reports.

It is important to try to classify the etiology of the lesions based on their nature, size, and location. Cerebral small vessel disease, the commonest cause of VCI, can be further categorized as cerebral amyloid angiopathy (CAA; main features are a lobar hemorrhage, cortical microbleeds, cortical siderosis; see modified Boston criteria^19^) and hypertensive microangiopathy (main features are lacunes, WMH, non-lobar hemorrhage, deep microbleeds^20^) or monogenetic causes such as CADASIL.^21^ Guidelines for these classifications and recommended ancillary tests are provided elsewhere.^16,18,19,22^ It should be noted, however, that assumptions on the etiology of vascular injury in individual patients based on lesion appearance are still imprecise.

### Linking cognitive decline with vascular brain lesions

In a patient with both cognitive impairment and vascular brain injury, the final step in the diagnosis VCI is to establish causality. This can be straightforward when there is a clear temporal relation between occurrence of a stroke and cognitive impairment or if the patient has a typical presentation of vascular subcortical injury, including impaired processing speed, executive dysfunction, gait impairment, and urinary incontinence. However, in many cases this can be challenging and many clinicians will recognize the large interindividual variability in the relation been the burden of (covert) vascular brain injury and cognitive symptoms. In these circumstances, the diagnosis is often based on expert opinion. Simply marking all patients with a high burden of vascular lesions as having VCI does not do justice to the high intersubject variability in the relation between lesion burden and cognitive performance. In particular, WMH are extremely common and there is no single threshold that reliably separates cognitively intact individuals from patients with VCI based on their lesion burden. Development of more reliable ways to translate brain imaging findings to cognitive symptoms in individual patients should therefore be a priority in VCI research.

Translating brain imaging findings to cognitive profiles of individual patients might be improved by taking lesion location into account.^23^ Traditionally, infarcts in the thalamus, corpus callosum, caudate nucleus, internal capsule, and left angular gyrus are considered strategic and small infarcts in these locations can cause major cognitive impairment.^5^ These insights, which were largely based on expert observations and small case series, are now corroborated and extended by a large multicenter study that generated the first comprehensive map of strategic infarct locations predicting post-stroke cognitive impairment.^10^ Lesion location may also be relevant for the cognitive impact of covert infarcts and WMH,^23^ but comprehensive, validated, location-based diagnostic tools for these lesion types are not yet available.

## Treatment

In people diagnosed with VCI, treatment strategies entail symptomatic treatment, optimizing quality of life and self-reliance, and modifying vascular risk.

### Symptomatic treatment

The general approach to treating cognitive symptoms and optimizing quality of life and self-reliance of the patient and their caretakers in VCI is similar as for other causes of dementia. Treatment for post-stroke cognitive impairment can include rehabilitation, primarily to provide the patient with insight in the nature of the deficits and develop adaptation strategies. Because there is insufficient evidence for a beneficial effect of acetylcholinesterase inhibitors or memantine in patients with VCI, guidelines do not recommend the use of these drugs.^22^ In case of mixed pathology, the use of acetylcholinesterase inhibitors or memantine can be considered following guidelines for the treatment of Alzheimer’s dementia, Lewy body dementia or Parkinson’s disease dementia. Neuropsychiatric symptoms such as anxiety or depression can be treated with medication if conservative measures are insufficiently effective, following general dementia guidelines.

### Treatment of vascular disease mechanisms and risk factors

If the patient has had a stroke or another cardiovascular event, secondary prevention guidelines for that event generally apply (see the recent American Heart Association and American Stroke Association (AHA/ASA) guidelines).^24^ In case of covert vascular brain lesions, adherence to primary vascular prevention guidelines is advised,^18,22^ as there currently is insufficient evidence to justify application of secondary prevention strategies (including the use of antiplatelet drugs) in such patients.^18,22^ If a patient meets the criteria for CAA and has an indication for using anticoagulation or platelet inhibitors, risks and benefits should be carefully weighed considering the high risk of intracerebral hemorrhage. Suggestions on how to deal with this dilemma and weigh the risks of thrombo-embolic versus hemorrhagic events are provided elsewhere.^25^

Hence, current treatment is still largely limited to general principles of primary and secondary cardiovascular risk management.^18,22^ Although this is clearly important, consideration of underlying cardiovascular disease mechanisms is likely of additional value.^21^ This calls for approaches to actually identify these mechanisms and develop targeted treatment.

### Future perspectives

In our view, the abovementioned limitations of current diagnostic criteria and dilemmas in clinical practice call for a fundamentally different approach to diagnosing VCI. This should be more dimensional, address interrelated—but not interchangeable—aspects such as cognitive impact of vascular brain injury, prognosis, and biological definitions of disease that can support targeted treatment. In the final section of this review, we will summarize promising developments in the field that can form the basis of such a new approach (Summarized in Box 2).

**Box 2.** Challenges in VCI assessment and possible solutions.

Dilemma’s and challenges	In development
Assessing severity of vascular brain injury in clinical practice is imprecise	- Quantitative assessment of lesion burden - Normative data according to age - Multimodal assessment of brain injury - Beyond visible lesions, e.g., diffusion MRI
Determining etiology and mechanisms of vascular brain injury is imprecise	- Toward a biological definition of VCI: markers for underlying disease processes and mechanisms
Vascular lesion burden relates poorly to cognitive functioning at an individual level	- Consider lesion burden but also location - Mapping of multiple lesion types - Integration with microstructural metrics and connectomics - Indicators of cognitive reserve / brain resilience

VCI: vascular cognitive impairment; MRI: magnetic resonance imaging.

### Assessing cognitive impact of vascular brain injury

**Figure 2.**
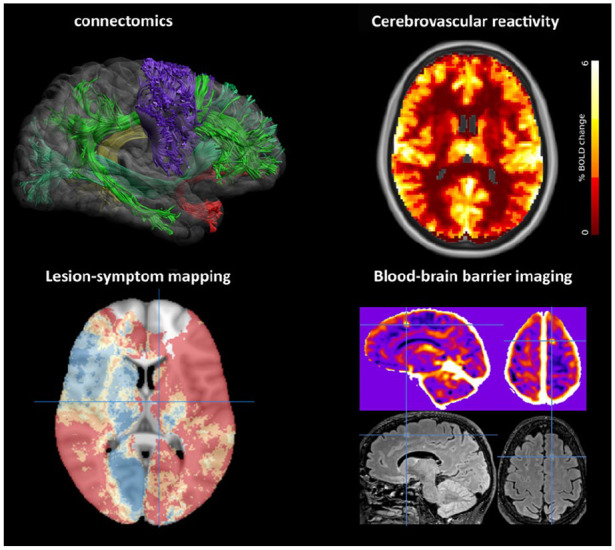
Emerging brain imaging techniques in VCI. Innovative brain imaging methods to improve detection of functional impact (left side of the figure) and underlying mechanisms (right) of vascular injury in VCI are developing rapidly. Several examples are shown. Connectomics: reconstruction of white matter tracts from diffusion-weighted imaging (image courtesy of Alberto De Luca, UMC Utrecht). Lesion-symptom mapping: brain vulnerability map derived from 2950 ischemic stroke patients, showing the predicted risk (dark blue: lowest risk; red: highest risk) of post-stroke cognitive impairment based on infarct location; crosshair indicates the left thalamus.^10^ Cerebrovascular reactivity: voxelwise reactivity maps derived from fMRI showing change in Blood-oxygen-level-dependent (BOLD) signal after hypercapnic stimulus (image courtesy of Hilde van den Brink, UMC Utrecht; SVDs@target consortium).^32^ Blood-brain barrier (BBB) imaging with Dynamic Contrast Enhanced (DCE) MRI (upper panel) showing a frontal BBB leakage hotspot (crosshairs). The corresponding FLAIR image (lower panel) shows a hyperintense lesion at the same site (image courtesy of Michael Thrippleton and Joanna Wardlaw, University of Edinburgh, SVDs@target consortium).

More than the type of vascular disease, it is the extent of the ensuing brain injury that determines cognitive impact. Brain imaging informs on this injury, but in current clinical practice much of the information contained in the images remains unused. Vascular lesions are primarily assessed visually, without use of proper normative data. In a memory clinic setting, such visual ratings explain little variance in cognitive functioning,^26^ limiting their diagnostic value to understand cognitive impact. Likewise, after ischemic stroke, crude infarct size is not a strong determinant of post-stroke cognitive impairment.^10^ It is likely that quantitative approaches have more diagnostic potential, particularly when information of different lesion types and atrophy is combined, not only considering lesion volumes, but also lesion distribution and location (Figure 2). The latter proves to be a strong determinant for post-stroke cognitive impairment^10^ and the same might also be true for other lesions, such as WMH. In addition, brain imaging can provide metrics of microstructural brain integrity in VCI beyond visible injury and information on brain connectomics.^27^ Microstructural diffusion MRI metrics like Peak Width of Skeletonized Mean Diffusivity^28^ (PSMD), free water diffusion,^29^ and MD median^30^ have shown to be stronger determinants of cognitive functioning in patients with vascular brain injury than all visible lesion types combined. Interestingly, there are also efforts to derive indices of brain resilience from connectivity measures extracted from diffusion or functional MRI.^31^ Integration of these different techniques might yield much more accurate individualized assessment of cognitive impact of vascular injury. Yet, clinical implementation requires important additional steps in technique and model development, harmonization, and validation.

### Prognostic models

VCI is associated with poor long-term clinical outcomes, including major adverse cardiovascular events and functional decline, not limited to cognitive functioning, but also involving behavioral changes and deterioration of gait.^5^ Yet, there is substantial interindividual variation in prognosis, depending among others on risk factor profile, nature of the underlying vascular disease, and co-morbidities. This variation provides challenges in clinical care, but also in research. The latter was reflected, for example, in the Secondary Prevention of Small Subcortical Strokes (SPS3) trial, where participants developed cognitive impairment at an annual rate of ~10%, but where average cognitive performance across the cohort remained stable over time.^33^ This indicates that trials in VCI that aim to use cognitive decline as an outcome may need to enrich their sample for patients at increased risk of cognitive decline. Risk scores to identify such patients with VCI are currently emerging.^34^ In clinical practice, establishing prognosis is important to inform patients, but also to guide treatment decisions. A quite common dilemma, also mentioned above, is how to balance risks of ischemic and hemorrhagic events in patients taking antithrombotic therapy who are found to have cerebral microbleeds. In the absence of clinical trials, we see strong data emerging from large multicenter observational studies,^35^ that can inform about individual risk of both ischemic and hemorrhagic events and may support such treatment decisions. Over the past decade there have been important initiatives for collaborative research in the small vessel disease and VCI field^36^ that may form the basis for development and validation of further prognostic scores.

### Biological definitions of disease processes

When the National Institute on Aging and Alzheimer’s Association published their Research Framework, proposing to define Alzheimer’s disease (AD) with biomarkers reflecting its underlying pathologic processes (i.e. beta amyloid deposition, pathologic tau, and neurodegeneration—ATN^37^), this created quite a stir in the VCI field. It was readily suggested that the “V” for vascular should be added to the framework.^38^ Of note, the rationale behind the ATN framework was that a biological rather than a syndromal definition of disease would support better understanding of mechanisms and—more importantly—that disease-modifying interventions must engage biologically defined targets and dementia does not denote a specific biological target.^37^ In light of this, if the VCI field would be invited to submit a “V” tomorrow, what biomarkers should then be included? In fact, we might even see the ATN framework as a wake-up call that should make us realize that current definitions of VCI contain few biologically defined targets that can be engaged with specific disease-modifying interventions. MRI markers such as WMH and diffusion metrics, for example, are clearly linked to VCI, but are primarily injury markers that can result of a multitude of biological processes and as such do not qualify as a treatment target, although they could serve as treatment outcomes. The pathophysiological chain from vascular risk factors to vascular brain injury is heterogeneous and includes many disease processes such as inflammation, coagulation cascade activation, endothelial dysfunction, and neurovascular unit dysfunction.^39^ Importantly, being able to pinpoint these disease processes could be a crucial step toward an individualized biological definition of VCI and offers opportunities to develop new treatments targeted at specific disease mechanisms. Approaches to pinpoint disease processes in VCI are currently being investigated, for example, blood-brain barrier imaging,^40^ neurovascular unit functional imaging,^32^ measures of cerebral blood flow,^32^ or inflammation^41^ (examples in Figure 2), but may by themselves not yet represent biological targets that can be engaged with specific drugs. Another interesting angle to pinpoint disease mechanisms is to identify specific pathologies such as arteriolosclerosis through patterns of injury on in vivo MRI using machine learning algorithms, trained with autopsy data.^42^ Finally, blood-based omics biomarkers may be of particular interest as well as rapid developments in the field of genetics of cerebrovascular disease that may deliver biological targets.^43^ Ultimately, it is expected that a more biological definition of the V in VCI will entail multiple components. Further developments in that field are eagerly awaited.

A final important point to consider is that optimal diagnostic approaches may differ according to the setting in which VCI criteria are to be used. For clinical practice, diagnostic clarity is essential, informing about the nature of the disease, how it explains the symptoms, what treatment options are available, and prognosis. Moreover, required diagnostic tools should be widely available. For research, more fine-grained criteria may be needed, particularly for selection of patients in trials. Initial trials with new disease modifying agents rely on careful selection of patients, where the disease process that is targeted should be present, be still at a stage when it is likely still modifiable, and where the primary clinical outcome that will be assessed is sufficiently likely to occur to be able to detect a treatment effect. In this setting, co-occurring other etiologies will often also be ruled out. This may require diagnostic tests that are not yet part of routine practice. Although findings in such highly selected patients may have limited generalizability to VCI at large, this approach is important because it is most likely to show proof of concept of novel treatments.

## Search strategy and selection criteria

For this narrative review on VCI diagnosis, PubMed was searched for articles published in English in the 10 years before 15 October 2021, using the terms “vascular”[Title] AND (“cognitive impairment”(All Fields) OR (“dementia”(MeSH Terms) OR “dementia”(All Fields) OR “dementias”(All Fields) OR “dementia s”(All Fields))). This returned 2223 results. Combined with ((((((diagnostic criteria) OR (guideline)) OR (criteria)) OR (statement)) OR (guidance)) OR (consensus)) OR (definition), this yielded 282 results. Titles and abstracts were screened by G.J.B. for relevance to the topics covered in this review. Further relevant publications were also taken from the authors’ records. In order to limit the number of citations, we refer to reviews where possible.
